# CD19-ReTARG^TPR^: A Novel Fusion Protein for Physiological Engagement of Anti-CMV Cytotoxic T Cells Against CD19-Expressing Malignancies

**DOI:** 10.3390/cancers17142300

**Published:** 2025-07-10

**Authors:** Anne Paulien van Wijngaarden, Isabel Britsch, Matthias Peipp, Douwe Freerk Samplonius, Wijnand Helfrich

**Affiliations:** 1 University of Groningen, Laboratory for Translational Surgical Oncology, Department of Surgery, University Medical Center Groningen, 9713 GZ Groningen, The Netherlands; a.p.van.wijngaarden@umcg.nl (A.P.v.W.); i.britsch@umcg.nl (I.B.); d.f.samplonius@umcg.nl (D.F.S.); 2 Department of Stem Cell Transplantation and Immunotherapy, Christian-Albrechts-Universität zu Kiel, Arnold-Heller Strasse 3, 24105 Kiel, Germany; m.peipp@med2.uni-kiel.de

**Keywords:** Immunotherapy, hematological cancer, CD19, TCR, CMV, antiviral immunity, BiTE, CAR T cell, AICD, CRS

## Abstract

**Background/Objectives**: The physiological activation of cytotoxic CD8^pos^ T cells (CTLs) relies on the engagement of the TCR/CD3 complex with cognate peptide-HLA class I (pHLA-I) on target cells, triggering cell lysis with appropriate cytokine release and minimized off-target toxicity. In contrast, current immunotherapies for CD19-expressing hematological malignancies, such as chimeric antigen receptor (CAR) T cells and bispecific T cell engagers (BiTEs)*,* bypass TCR/pHLA interactions, resulting in CTL hyperactivation and excessive cytokine release, which frequently cause severe immune-related adverse events (irAEs). Thus, there is a pressing need for T cell-based therapies that preserve physiological activation while maintaining antitumor efficacy. **Methods**: To address this, we developed CD19-ReTARG^TPR^, a novel fusion protein consisting of the immunodominant cytomegalovirus (CMV) pp65-derived peptide TPRVTGGAM (TPR) covalently presented by a soluble HLA-B*07:02/β2-microglobulin complex fused to a high-affinity CD19-targeting Fab antibody fragment. The treatment of CD19-expressing cancer cells with CD19-ReTARG^TPR^ makes them recognizable for pre-existing anti-CMV_pp65_ CTLs via physiological TCR-pHLA engagement. **Results**: Our preclinical data demonstrate that CD19-ReTARG^TPR^ efficiently redirects anti-CMV CTLs to eliminate CD19-expressing cancer cells, including both established cell lines and primary chronic lymphocytic leukemia (CLL) cells. Unlike CD19-directed CAR T cells or the CD19/CD3 BiTE blinatumomab, CD19-ReTARG^TPR^ mediated *robust* cytotoxic activity without *triggering* supraphysiological cytokine *release*. Importantly, this approach retained efficacy even against cancer cells with low CD19 expression. **Conclusions**: In summary, we provide a robust proof-of-concept study and show that CD19-ReTARG^TPR^ offers a promising alternative strategy for T cell redirection, enabling the selective and effective killing of CD19-expressing malignancies while minimizing cytokine-driven toxicities through physiological CTL activation pathways.

## Simple Summary

Current treatments for certain blood cancers, like CAR T cells and BiTEs, can overactivate the immune system and cause serious side effects. This study introduces a new therapy called CD19-ReTARG^TPR^, which works by helping the body’s existing virus-fighting T cells recognize and attack cancer cells in a more natural and controlled way. It achieves this by attaching a small piece of a common virus (CMV) to a targeting molecule that binds to cancer cells, allowing T cells to kill the cancer without excessive immune reactions. In lab tests, this approach effectively killed cancer cells, even those with low levels of the target marker (CD19), and caused fewer harmful side effects compared to current therapies. This new strategy could lead to safer and more precise immunotherapies for patients with CD19-positive blood cancers.

## 1. Introduction

Adoptive immunotherapies have transformed the treatment landscape for B cell malignancies, particularly those expressing CD19. However, many of these approaches, including chimeric antigen receptor (CAR) T cells and bispecific T cell engagers (BiTEs), exert their cytotoxic effects through TCR-independent mechanisms. While effective in tumor clearance, these strategies frequently induce uncontrolled T cell hyperactivation and substantial proinflammatory cytokine release, contributing to serious immune-related adverse events (irAEs), such as cytokine release syndrome (CRS) and immune effector cell-associated neurotoxicity syndrome (ICANS). Moreover, continuous hyperactivation can lead to activation-induced cell death (AICD), limiting T cell persistence and diminishing long-term efficacy [1,2].

In contrast, physiological CTL responses rely on the interaction of the T cell receptor (TCR) with cognate peptide-HLA class I (pHLA-I) complexes on target cells. This mode of activation ensures precise target cell lysis while tightly regulating cytokine production and minimizing bystander damage. Immunotherapeutic platforms that preserve this natural axis of T cell activation may enable effective tumor clearance with reduced systemic toxicity.

CTLs specific for ubiquitous persistent viruses, particularly human cytomegalovirus (HCMV), constitute a sizable and functionally competent effector pool that remains underexploited in current T cell redirection strategies. CMV-seropositive individuals harbor large populations of so-called inflationary CD8^pos^ T cells specific for immunodominant CMV antigens, such as the pp65-derived HLA-B*07:02-restricted peptide TPRVTGGAM (TPR). Anti-CMV T cells can constitute more than 10% of the peripheral CTL repertoire and tend to increase with age [3]. They display an effector–memory phenotype [4,5,6], retain robust cytotoxic function, and exhibit broad tissue homing, including infiltration into tumor microenvironments [7].

Importantly, in patients with chronic lymphocytic leukemia (CLL), a disease characterized by widespread T cell exhaustion and dysfunction, anti-CMV CTLs retain their functionality, unlike autologous tumor-reactive T cells [8]. This observation underscores the potential of redirecting CMV-specific CTLs to overcome immune evasion mechanisms in hematologic malignancies.

In recent years, several strategies have been developed that aim to redirect the unique cytotoxic potential of inflationary anti-CMV CD8^pos^ T cells to selectively eliminate cancer cells. The majority of these rely on intact antigen processing and presentation machinery, while it is well known that these are frequently modified or defective in cancer cells [9]. We engineered fusion protein CD19-ReTARG^TPR^, a novel preassembled CMV peptide/HLA-I T cell engager designed to mimic CMV antigen presentation on CD19-expressing cancer cells. CD19-ReTARG^TPR^ comprises the TPR peptide covalently bound to a soluble HLA-B*07:02/β2-microglobulin complex, which is genetically fused to a high-affinity CD19-specific Fab antibody fragment. Treatment with CD19-ReTARG^TPR^ selectively coats CD19-expressing malignant B cells with its TPR–pHLA complex, thereby allowing physiological engagement by CMV-specific CTLs with cognate TCRs. This mode of action circumvents the requirement of intact antigen processing and the presence of HLA.

Here, we describe the preclinical in vitro evaluation of CD19-ReTARG^TPR^ using both established cell lines and primary patient-derived CLL cells. Our results demonstrate that CD19-ReTARG^TPR^ effectively redirects anti-CMV CTLs to selectively eliminate CD19-expressing cancer cells without inducing supraphysiological cytokine responses. Importantly, CD19-ReTARG^TPR^ retained efficacy even against cancer cells with low CD19 expression. These findings support CD19-ReTARG^TPR^ as a promising, physiologically tuned immunotherapeutic candidate for the treatment of CD19-expressing malignancies. Together, we provide a fundamental feasibility study on redirecting anti-CMV T cells to eliminate CD19^pos^ cancer B cells.

## 2. Materials and Methods

### 2.1. Antibodies and Reagents

The following fluorochrome-conjugated monoclonal antibodies (mAbs) against human antigens were used: PerCP-Cy5.5-conjugated anti-CD3 (clone OKT-3) and APC-conjugated anti-CD8 (clone RPA-T8) (both from eBioscience, San Diego, CA, USA); PE-conjugated anti-CD3, FITC-conjugated anti-CD8, APC-conjugated anti-CD19, and FITC-conjugated anti-CD20 (all from ImmunoTools, Friesoythe, Germany); APC-conjugated anti-HLA-B7 (clone BB7.1, BioLegend Europa, Amsterdam, The Netherlands); anti-2a peptide (Sigma-Aldrich, Zwijndrect, The Netherlands); and Alexa Fluor 647-conjugated anti-mouse IgG (Invitrogen, Waltham, MA, USA). The following recombinant antibodies were used: blinatumomab (Invitrogen), tafasitamab (MOR208), and rituximab (AntibodySystem, Paris, France). Additional reagents included FITC-labeled Annexin V (ImmunoTools), APC-conjugated HLA-B*07:02/TPRVTGGAM (TPR) streptamer (IBA Lifesciences, Göttingen, Germany), propidium iodide (PI) (Invitrogen), Vybrant DiD, and CFSE CellTrace Far Red (ThermoFisher, Waltham, MA, USA). Cytokine detection was performed using an IFN-γ ELISA kit (ThermoFisher) and a human cytokine antibody array (42 targets; Abcam, Boston, MA, USA, ab133997).

### 2.2. Cell Lines and Transfectants

Human CD19-positive hematologic cancer cell lines (SEM, Ramos, JeKo-1, Granta-519, Wil2-S, Namalwa, Kasumi-1, Z138, and HG-3) and CD19-negative lines (K562, CEM, and CHO) were obtained from ATCC (Manassas, VA, USA). Cells were maintained in RPMI-1640 or DMEM (Lonza, Geleen,The Netherlands), supplemented with 10% fetal calf serum (FCS), at 37 °C in a humidified 5% CO_2_ atmosphere. Stable CD19-expressing CHO and CEM cells were generated via lipofection (Fugene-HD, Promega BNL, Leiden, The Netherlands) with a plasmid encoding human CD19 cDNA (Origene Technologies GmbH, Herford, Germany), followed by clonal selection via limiting dilution. CD19 expression was confirmed by flow cytometry (Guava easyCyte 6-2L using InCyte 3.2 software). CD19 antigen density was estimated using the BD QuantiBrite Kit as per manufacturer’s protocol. OvCAR3.pp65 cells were generated by lipofection with plasmid pCMV6-pp65 encoding full-length CMVpp65 protein (Origene) followed by clonal selection via limiting dilution.

### 2.3. Primary Patient-Derived B-CLL Cells

Peripheral blood mononuclear cells (PBMCs) were isolated from heparinized blood of five B-cell chronic lymphocytic leukemia (B-CLL) patients. Primary patient-derived cancer cells were cultured in RPMI-1640 supplemented with 10% FCS at 37 °C in a humidified 5% CO_2_ atmosphere. The study was conducted in accordance with ethical standards and with approval of the Ethics Committee of the Kiel University (PV5777, 26 April 2016).

### 2.4. Ex Vivo Expansion of Anti-CMV_pp65_ CD8^pos^ T Cells

Anti-CMV CD8^pos^ T cells were expanded as described previously [10,11]. In short, peripheral blood mononuclear cells (PBMCs) from HLA-B*07:02^pos^/CMV-seropositive individuals were purchased from CTL-Europe GmbH (Rutesheim, Germany) and stimulated with CMV pp65-derived peptide TPRVTGGAM (TPR) for 4 d. Subsequently, TPR-stimulated PBMCs were harvested, resuspended in fresh X-VIVO15 medium (Lonza) supplemented with 50 IU/mL rh IL2, and cultured for an additional 4 d. Restimulation was performed by culturing TPR/IL2-stimulated PBMCs on a feeder layer of OvCAR3.pp65 cells at an effector-to-target (E:T) cell ratio of 20:1. Restimulated anti-CMV_pp65_ T cells were then resuspended in X-VIVO15 medium supplemented with 100 IU/mL rh IL2. Medium was refreshed every 2–3 days. Stimulation of anti-CMV_pp65_ CD8^pos^ T cells was repeated every 14 d. Percentage of HLA-B*07:02-TPR-specific T cells was assessed using streptamer staining by flow cytometry, which indicated that this stimulation protocol yielded up to 60% HLA-B*07:02-TPR-streptamer^pos^ CD8^pos^ T cells.

### 2.5. Construction, Production, and Purification of CD19-ReTARG^TPR^

Construction, production, and purification of CD19-ReTARG^TPR^ were similar to previous reports [11]. CD19-ReTARG^TPR^ consists of CMV pp65 peptide TPRVTGGAM, β2 microglobulin, and a truncated HLA-B*07:02 heavy chain lacking its transmembrane and intracellular domains. The stability of CD19-ReTARG^TPR^ was enhanced by C-terminal fusion of TPR to the linker sequence G**C**GGSGGGGSGGGGS, which was engineered to contain a cysteine residue (in bold) inserted in the α1 domain HLA-B*07:02 heavy chain that promotes the formation of an intramolecular disulfide bridge. Using a flexible linker, the HLA-I-α chain was genetically fused to the anti-CD19 Fab antibody domain containing VH-VL gene segments of Tafasitamab (MOR208). An analogous ReTARG variant with irrelevant target specificity, designated Mock-ReTARG^TPR^, was constructed by exchanging the anti-CD19 Fab for an anti-EpCAM Fab domain as described previously [11]. The cDNAs encoding the respective fusion proteins were synthesized and then cloned into the eukaryotic expression plasmid pcDNA3.1-hygro by Genscript (Rijswijk, The Netherlands) and then transfected by lipofection (Fugene-HD, Promega) into HEK293AD cells. After 7 d, conditioned cell culture supernatants were harvested and cleared by centrifugation (3000× *g*, 30 min). ReTARGs were purified using a Capture Select^TM^ CH1-XL column (ThermoFisher) connected to an ÄKTA Start chromatography system (GE Healthcare Life Sciences, Eindhoven, The Netherlands). Purified proteins were diluted in PBS (1 mg/mL) and stored at −20 °C.

### 2.6. SDS-PAGE Analysis

Purified CD19-ReTARG^TPR^ (2 μg/lane) was analyzed by SDS-PAGE using 10% acrylamide gels under reducing and non-reducing conditions, followed by Coomassie brilliant blue staining.

### 2.7. CD19 Binding Assays

Binding of CD19-ReTARG^TPR^ and Mock-ReTARG^TPR^ to CD19-expressing cells was evaluated by flow cytometry. In short, cells were incubated with increasing concentrations (10–10,000 ng/mL) of ReTARG fusion protein at 4° C for 45 min. Bound fusion proteins were detected using an APC-conjugated anti-HLA-B7 antibody or anti-2a peptide antibody followed by Alexa Fluor 647-conjugated secondary antibody.

### 2.8. In Vitro Cytotoxicity Assays

CD19-positive and CD19-negative cell lines, as well as primary CLL patient-derived cells, were co-cultured with TPR/IL2-expanded PBMCs at indicated effector-to-target (E:T) cell ratios for 24 h and treated with CD19-ReTARG^TPR^ or Mock-ReTARG^TPR^. For comparison, cocultures were treated with blinatumomab (5 ng/mL) or second-generation CD19-CAR T cells (FMC63-CD19 scFv with CD3ζ and 4-1BB domains, kindly provided by Prof. Dr. E. Bremer, UMC Groningen, Groningen, The Netherlands). Apoptotic cancer cell death was measured after 24 h by Annexin V/PI staining and flow cytometry.

### 2.9. Assessment of Activation-Induced Cell Death (AICD)

Effector cells (TPR/IL-2-expanded PBMCs with CD19-ReTARG^TPR^, PBMCs with blinatumomab, or CD19-CAR T cells) were co-cultured with CD19-positive or CD19-negative target cells at the indicated E:T ratios for 24 h. Effector cell viability was assessed via PI staining and flow cytometry.

### 2.10. Cytokine Secretion Analysis

Supernatants from co-cultures of effector and target cells were collected after 24 h*.* IFNγ levels were quantified using ELISA (ThermoFisher). Alternatively, cytokine profiles were evaluated using a 42-plex human cytokine antibody array (Abcam).

### 2.11. Statistical Analysis

Data were analyzed using GraphPad Prism version 10.2.3 (GraphPad Software). Statistical significance was assessed by unpaired two-tailed Student’s *t*-test or one-/two-way ANOVA, followed by appropriate multiple comparisons tests. *p*-values were considered significant as follows: * *p* < 0.05, ** *p* < 0.01, *** *p* < 0.001.

## 3. Results

### 3.1. Construction, Production, and Purification of CD19-ReTARG^TPR^

**Figure 1:**
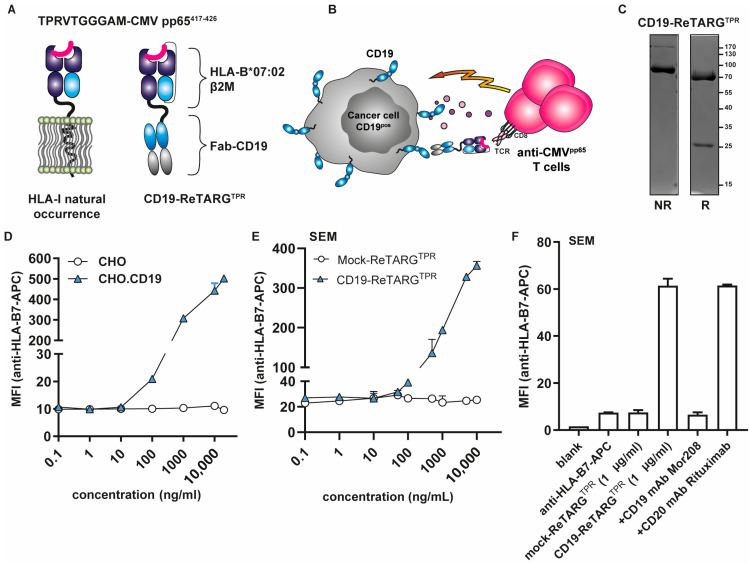
CD19-ReTARG^TPR^ selectively binds to CD19^pos^ cancer cells. (**A**) Schematic illustration of the CD19-ReTARG^TPR^ (**right**) fusion protein compared to a natural peptide-HLA class I complex on human cells (**left**). (**B**) Proposed mechanism of action of CD19-ReTARG^TPR^. (**C**) SDS-PAGE analysis of CD19-ReTARG^TPR^ stained with Coomassie brilliant blue under non-reducing (NR; lane 1) and reducing (R; lane 2) conditions. The uncropped blots are shown in the Supplemental Materials. (**D**) Dose-dependent binding of CD19-ReTARG^TPR^ to parental CHO and CHO.CD19 cells. (**E**) Dose-dependent binding of CD19-ReTARG^TPR^ (and Mock-ReTARG^TPR^) to CD19^pos^ B-ALL SEM cells. (**F**) Binding of CD19-ReTARG^TPR^ (1 μg/mL) to CD19^pos^ SEM cells is abolished in the presence of an excess of the competing antibody, anti-CD19 mAb MOR208. Flow cytometry was used for panels (**D**–**F**). Graphs show two technical replicates (mean ± SD).

A schematic illustration of the CD19-ReTARG^TPR^ fusion protein and its proposed mode of action are shown in Figure 1A,B, respectively. CD19-ReTARG^TPR^ appears to be stable, and an SDS-PAGE analysis of purified CD19-ReTARG^TPR^ indicated an apparent molecular weight (MW) of ~95 kDa, which was in accordance with its calculated MW of 96.9 kDa (Figure 1C). Under reducing conditions, CD19-ReTARG^TPR^ migrated as two protein bands of ~70 kDa and ~25 kDa. The 25 kDa protein band represents the light chain of the CD19-targeting antibody Fab domain.

### 3.2. CD19-ReTARG^TPR^ Selectively Binds to CD19^pos^ Cancer Cells

CD19-ReTARG^TPR^ selectively bound to CHO.CD19 cells with no detectable binding to parental CHO cells (Figure 1D). Moreover, CD19-ReTARG^TPR^ bound dose-dependently to CD19^pos^ B-ALL SEM cells, whereas Mock-ReTARG^TPR^ failed to do so (Figure 1E). The CD19-selective binding of CD19-ReTARG^TPR^ was abrogated in the presence of a surplus of the competing antibody, CD19 mAb MOR208 (Figure 1F).

### 3.3. CD19-ReTARG^TPR^ Selectively Redirects the Cytotoxic Activity of Anti-CMV_pp65_ CD8^pos^ T Cells Towards CD19^pos^ Cancer Cell Lines and Patient-Derived CLL Cells

**Figure 2:**
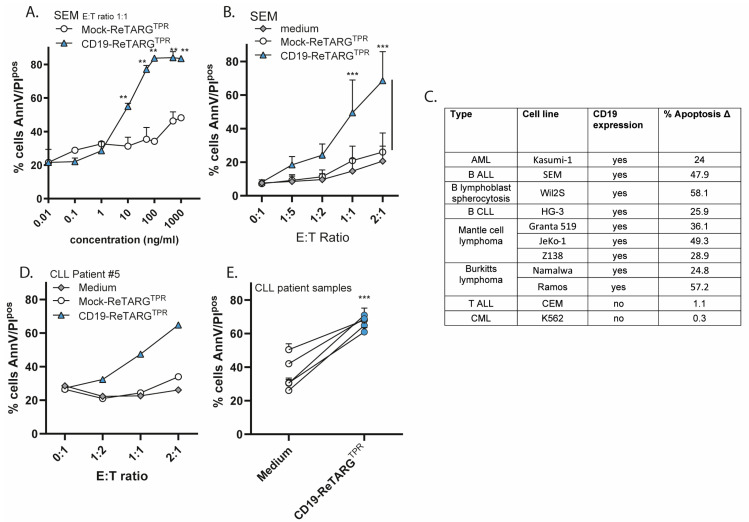
CD19-ReTARG^TPR^ selectively redirects the cytotoxic activity of anti-CMV_pp65_ CD8^pos^ T cells towards CD19^pos^ cancer cell lines and patient-derived CLL cells. (**A**) Cytotoxic capacity of anti-CMV CD8^pos^ T cells towards CD19^pos^ SEM cells (E:T cell ratio = 1:1) treated with increasing concentrations (0–1000 ng/mL) of CD19-ReTARG^TPR^ or Mock-ReTARG^TPR^. (**B**) Cytotoxic capacity of anti-CMV CD8^pos^ T cells towards CD19^pos^ SEM at increasing E:T cell ratios treated with CD19-ReTARG^TPR^ or Mock-ReTARG^pp65^ (both 100 ng/mL). (**C**) Cytotoxic capacity of anti-CMV CD8^pos^ T cells towards a range of CD19^pos^ or CD19^neg^ cell lines (including AML, CML, B-ALL, B lymphoblast spherocytosis, B-CLL, Mantle cell lymphoma, Burkitt’s lymphoma, and T-ALL; E:T cell ratio = 2:1) treated with CD19-ReTARG^TPR^ (100 ng/mL). Apoptosis (%) is shown as Δ(CD19-ReTARG^TPR^-anti-CMV_pp65_ CD8^pos^ T cells). (**D**) Cytotoxic capacity of anti-CMV CD8^pos^ T cells towards CD19^pos^ cancer B cells from a CLL patient (#5) at increasing E:T cell ratios treated with CD19-ReTARG^TPR^ or Mock-ReTARG^TPR^ (both 100 ng/mL). (**E**) Cytotoxic capacity of anti-CMV CD8^pos^ T cells towards CD19^pos^ cancer cells from 5 CLL patients (E:T cell ratio = 2:1). Apoptotic cancer cell death was assessed using Annexin V/PI staining after 24 h. Graphs A–E: *n* = 3 (two technical replicates); mean ± SD are shown. Statistical analysis in A,B,E was performed using unpaired *t*-test (Mock-ReTARG^TPR^ versus CD19-ReTARG^TPR^) (** *p* < 0.01,*** *p* < 0.001).

**Figure A1:**
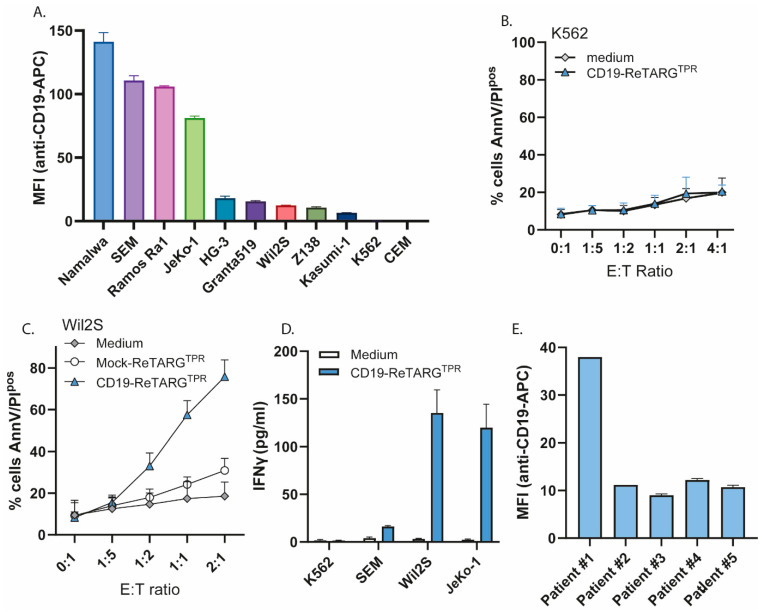
—CD19-ReTARG^TPR^ selectively redirects the cytotoxic activity of anti-CMV_pp65_ CD8^pos^ T cells towards CD19^pos^ cancer cell lines and patient-derived CLL cells. (**A**) CD19 cell surface expression on a panel of CD19-expressing or CD19-negative cell lines (including AML, CML, B-ALL, B lymphoblast spherocytosis, B-CLL, Mantle cell lymphoma, Burkitt’s lymphoma, and T-ALL). (**B**) Cytotoxic capacity of anti-CMV_pp65_ CD8^pos^ T cells towards CD19-negative K562 cells at increasing E:T cell ratios treated with CD19-ReTARG^TPR^ (100 ng/mL). Apoptotic cancer cell death was assessed using Annexin V/PI staining after 24 h. (**C**) Capacity of CD19-ReTARG^TPR^ (100 ng/mL) to activate anti-CMV_pp65_ CD8^pos^ T cells in the presence of K562. (**D**) Capacity of CD19-ReTARG^TPR^ to activate anti-CMV CD8^pos^ T cells in the presence of CD19-negative cell line or CD19-expressing cell lines, SEM, JeKo-1, and Wil2S (E:T cell ratio = 1:1). Conditioned culture media were collected after 24 h, and T cell-secreted IFNγ was quantified by ELISA. (**E**) CD19 expression on primary (CLL) patient-derived cancer B cells. Graph –E *n* = 3 (two technical replicates); mean ± SD are shown.

CD19-ReTARG^TPR^ selectively redecorated the SEM CD19^pos^ human B-ALL cell line and redirected the cytotoxic activity of anti-CMV CD8^pos^ T cells towards these cells in both a dose-dependent (Figure 2A) and effector-to-target (E:T) ratio-dependent manner (Figure 2B). Of note, in these in vitro experiments, there is an HLA mismatch of target cancer cells and effector anti-CMV T cells, which may explain a slight baseline difference in apoptosis. However, the level of specific killing in the presence of CD19-ReTARG^TPR^ and anti-CMV CTLs significantly exceeds background levels. CD19-ReTARG^TPR^ facilitated the potent elimination of a broad range of CD19^pos^ hematologic cancer cell lines of diverse origins (Figure 2C and Figure A1A,C) by anti-CMV CD8^pos^ T cells, while CD19^neg^ cell lines such as K562 and CEM remained unaffected (Figure 2C and Figure A1B). CD19-ReTARG^TPR^ also activated anti-CMV CD8^pos^ T cells, as evidenced by the increased secretion of the proinflammatory cytokine IFNγ (Figure A1D). Importantly, CD19-ReTARG^TPR^ mediated the robust elimination of primary CD19^pos^ cancer cells derived from five patients with chronic lymphocytic leukemia (CLL) (Figure 2D,E and Figure A1E). Together, these findings demonstrate that CD19-ReTARG^TPR^ efficiently redirects the cytotoxic capacity of anti-CMV CD8^pos^ T cells to selectively lyse CD19^pos^ cancer B cells.

### 3.4. CD19-ReTARG^TPR^ Retains Efficacy Against Cancer Cells with Low CD19 Expression

**Figure 3:**
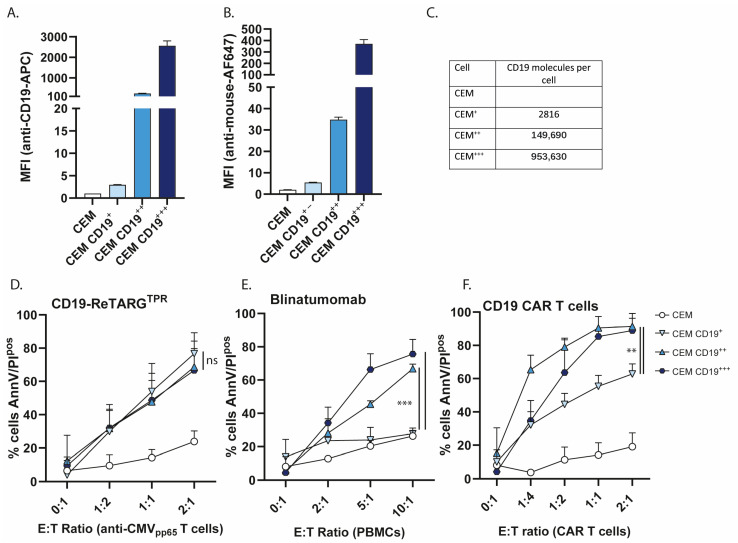
CD19-ReTARG^TPR^ retains efficacy against cancer cells with low CD19 expression. (**A**) CEM cell surface-expressed CD19 after transfection with the human CD19 plasmid and culturing of single cell clones. Cells were categorized as CD19^neg^, low (CD19^+^), intermediate (CD19^++^), and high (CD19^+++^). (**B**) Binding of CD19-ReTARG^TPR^ (1 μg/mL) to CD19^neg^ or CD19-expressing CEM cells. (**C**) Flow cytometric analysis of CD19 antigen density, semi-quantitatively determined using the BD Quantibrite kit. (**D**) Cytotoxic capacity of anti-CMV CD8^pos^ T cells towards CD19^neg^ or CD19-expressing CEM cells at increasing E:T cell ratios treated with CD19-ReTARG^TPR^ (100 ng/mL). (**E**) Cytotoxic capacity of PBMCs towards CD19^neg^ or CD19-expressing CEM cells at increasing E:T cell ratios treated with blinatumomab (5 ng/mL). (**F**) Cytotoxic capacity of CD19-CAR T cells towards CD19^neg^ or CD19-expressing CEM cells at increasing E:T cell ratios. For C,D, and E, apoptotic cancer cell death was assessed using Annexin V/PI staining after 24 h. Graphs (**A** + **B**) show two technical replicates (mean ± SD). Graphs (**D**–**F**): *n* = 3 (two technical replicates); mean ± SD are shown. Statistical analysis in C–E was performed using one-way ANOVA (CEM CD19^+^ vs. CEM CD19^++^ or CEM CD19^+++^) (ns = non-significant, ** *p* < 0.01, *** *p* < 0.001).

Target antigen downregulation is a well-documented resistance mechanism observed in both CAR T cell and BiTE therapies [12,13,14,15]. Therefore, we reasoned that CD19-ReTARG^TPR^ could be an effective strategy for eliminating cancer cells with low CD19 expression. To this end, we developed a model cell line panel in which we genetically engineered the CEM CD19^neg^ cell line to express varying levels of CD19. Single-cell clones were selected to stably express low (CD19^+^), intermediate (CD19^++^), and high (CD19^+++^) levels of CD19 (CD19 expression is shown in Figure 3A and binding of CD19-ReTARG^TPR^ is shown in Figure 3B). CD19 antigen density was estimated using the BD Quantibrite kit, and it indicated that the CD19 densities of CEM^+^, CEM^++^, and CEM^+++^ were ~2800, ~150,000, and ~950,000 molecules per cell, respectively (Figure 3C). Of note, it was reported that when the CD19 density falls below ~2000 molecules per cell, it significantly affects the anticancer efficacy by CAR T cells [16]. We then compared the capacity of CD19-ReTARG^TPR^ to eliminate the respective CEM cell line clones with CD19/CD3 BiTE blinatumomab and CD19 CAR T cells. Interestingly, CD19-ReTARG^TPR^ efficiently enabled anti-CMV CD8^pos^ T cells to lyse CEM CD19 cells irrespective of the level of CD19 expression (Figure 3D). In contrast, blinatumomab failed to mediate the elimination of CEM CD19^+^ cells (Figure 3E). Similarly, while CD19-CAR T cells effectively lysed CEM CD19^++^ and CEM CD19^+++^ (up to >90%), the lysis of CEM CD19^+^ was only ~60% (Figure 3F). Together, these data indicate that cancer cells with low CD19 expression remain sensitive to elimination by CD19-ReTARG^TPR^-redirected anti-CMV CD8^pos^ T cells.

### 3.5. CD19-ReTARG^TPR^ Induces Effective Lysis of CD19-Expressing Cancer B Cells with Reduced Proinflammatory Cytokine Release Compared to Blinatumomab and CD19 CAR T Cells

BiTEs and CAR T cells mediate cytotoxicity against CD19-expressing cells via TCR-independent mechanisms. While these approaches are effective in tumor eradication, they are frequently associated with excessive T cell activation and robust proinflammatory cytokine release, leading to immune-related adverse events (irAEs), including cytokine release syndrome (CRS) [16], which can limit their clinical applicability. In contrast, CD19-ReTARG^TPR^ engages T cells through a physiological, TCR/pHLA-I-restricted mechanism, which we hypothesized would result in reduced cytokine release.

**Figure 4:**
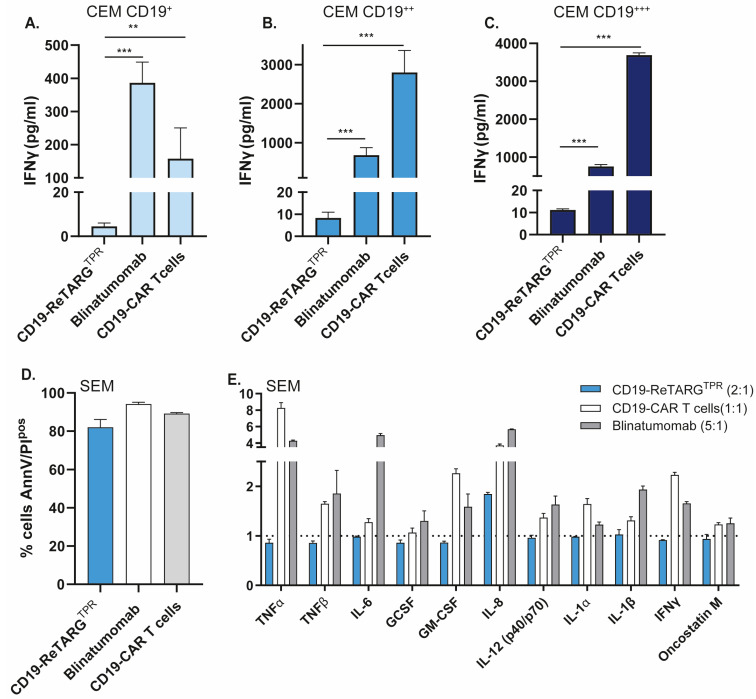
CD19-ReTARG^TPR^ induces effective lysis of CD19-expressing cancer B cells with reduced proinflammatory cytokine release compared to blinatumomab and CD19 CAR T cells. **A**–**C**: Comparison of T cell-secreted IFNγ levels after treatment of CEM CD19^+^ (**A**), CEM CD19^++^ (**B**), and CEM CD19^+++^ (**C**) by CD19-ReTARG^TPR^ (+ anti-CMV CD8^pos^ T cells, E:T = 2:1), blinatumomab (+ PBMCs, E:T = 5:1), and CD19-CAR T cells (E:T = 1:1). Conditioned culture media were collected after 24 h, and T cell-secreted IFNγ was quantified by ELISA. (**D**) Cytotoxic capacity of CD19-ReTARG^TPR^ (+ anti-CMV_pp65_ CD8^pos^ T cells, E:T = 2:1), blinatumomab (+ PBMCs, E:T = 5:1), and CD19-CAR T cells (E:T = 1:1) to eliminate SEM B-ALL cell line. Apoptotic cancer cell death was assessed using Annexin V/PI staining after 24 h. (**E**) Conditioned culture media were collected after 24 h, and T cell-secreted proinflammatory cytokines were quantified using a cytokine array. Graphs (**A**–**C**) *n* = 3 with two technical replicates (mean ± SD). Statistical analysis in (**A**–**C**) was performed using one-way ANOVA (CD19-ReTARG^TPR^ vs. blinatumomab or CD19-CAR T cells) (** *p* < 0.01, *** *p* < 0.001).

**Figure A2:**
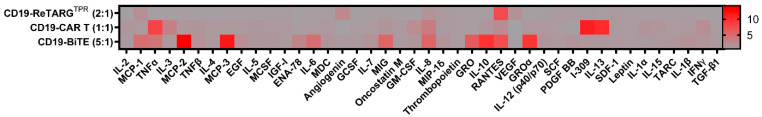
CD19-ReTARG^TPR^ induces effective lysis of CD19-expressing cancer B cells with reduced proinflammatory cytokine release compared to blinatumomab and CD19 CAR T cells. Heatmap of cytokine array comparing T cell-secreted IFNγ levels after treatment of SEM by CD19-ReTARG^TPR^ (+ anti-CMV CD8^pos^ T cells, E:T = 2:1), blinatumomab (+ PBMCs, E:T = 5:1), and CD19-CAR T cells (E:T = 1:1). Conditioned culture media were collected after 24 h.

To evaluate this, we quantified IFNγ levels during cytotoxicity assays and found that CD19-ReTARG^TPR^ treatment induced IFNγ secretion that was approximately 100-fold lower compared to that of blinatumomab and up to 400-fold lower relative to CD19 CAR T cells (Figure 4A–C). Furthermore, although CD19-ReTARG^TPR^, blinatumomab, and CD19 CAR T cells induced comparable lysis of B-ALL SEM cells (Figure 4D), CD19-ReTARG^TPR^ elicited significantly lower levels of secretion of several core proinflammatory cytokines involved in CRS, most notably TNFα, TNFβ, and IL6 (Figure 4E and Figure A2).

Collectively, these findings demonstrate that CD19-ReTARG^TPR^ achieves effective cytolytic activity against CD19-expressing malignant B cells while inducing substantially lower levels of cytokine release compared to current TCR-independent redirection strategies, highlighting its potential to reduce the risk of cytokine-mediated toxicities.

### 3.6. CD19-ReTARG^TPR^ Induces Minimal Activation-Induced Cell Death in Redirected Anti-CMV CD8^pos^ T Cells

**Figure 5:**
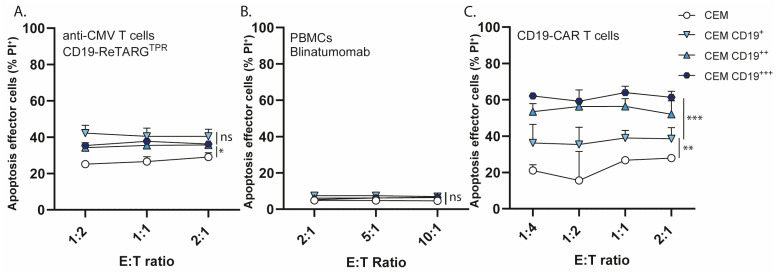
CD19-ReTARG^TPR^ induces minimal activation-induced cell death in redirected anti-CMV CD8^pos^ T cells. (**A**) Apoptosis of effector anti-CMV_pp65_ CD8^pos^ T cells after co-culture with CEM, CEM CD19^+^, CEM CD19^++^, or CEM CD19^+++^ cells at increasing E:T cell ratios treated with CD19-ReTARG^TPR^ (100 ng/mL). (**B**) Apoptosis of PBMCs after co-culture with CEM, CEM CD19^+^, CEM CD19^++^, or CEM CD19^+++^ cells at increasing E:T cell ratios treated with blinatumomab (5 ng/mL). (**C**) Apoptosis of CD19-CAR T cells after co-culture with CEM, CEM CD19^+^, CEM CD19^++^, or CEM CD19^+++^ cells at increasing E:T cell ratios. Effector cell death was assessed using PI staining after 24 h. Graphs (**A**–**C**): *n* = 3 (two technical replicates); mean ± SD are shown. Statistical analysis in (**A**–**C**) was performed using one-way ANOVA (CEM vs. CEM CD19^+^, CEM CD19^++^, or CEM CD19^+++^) (ns = non-significant, * *p* < 0.05,** *p* < 0.01, *** *p* < 0.001).

AICD is an essential homeostatic mechanism that ensures the timely termination of immune responses following antigen clearance. Previous studies have shown that CAR T cells, due to their uncontrolled tendency for non-physiological hyperactivation, are particularly susceptible to AICD [17,18], leading to reduced persistence and diminished therapeutic efficacy, while moderate signaling avoids this issue. We investigated whether anti-CMV T cells, upon activation by CD19-ReTARG^TPR^, were susceptible to AICD and assessed the fate of CMV CD8^pos^ T cells (Figure 5A), PBMCs (Figure 5B), and CD19 CAR T cells (Figure 5C), after target cell lysis. The results indicate that CD19 CAR T cells undergo apoptosis upon target cell lysis, and that the level of apoptosis induced appears to be correlated with CD19 expression on target cells, as CD19 CAR T cell apoptosis is more elevated upon treatment of CEM CD19^+++^ (Δ apoptosis ~40%) compared to CEM CD19^++^ and CEM CD19^+^ (Δ apoptosis ~20–30%). In contrast, anti-CMV CD8^pos^ T cells were only minimally affected (Δ apoptosis max ~10%) by target cell lysis, and no detectable apoptosis was observed in PBMCs. Together, these findings show that the redirection of anti-CMV CD8^pos^ T cells by CD19-ReTARG^TPR^ induces minimal AICD in effector cells.

## 4. Discussion

Current immunotherapies targeting CD19-expressing hematological malignancies, such as CAR T cells and BiTEs, inherently bypass cognate TCR/pHLA-I signaling and consequently induce the non-physiological hyperactivation of T cells. Typically, this leads to excessive proinflammatory cytokine release and reduced T cell longevity due to AICD [17,19].

In this study, we report on the development and preclinical evaluation of CD19-ReTARG^TPR^, a novel fusion protein that selectively binds to CD19-expressing cancer cells, resulting in their rapid elimination by pre-existing anti-CMV_pp65_ CTLs via physiological TCR/pHLA engagement. In vitro, CD19-ReTARG^TPR^ induced potent and selective lysis across a range of CD19-expressing cancer cell lines, as well as primary CLL cells derived from patients, without provoking excessive cytokine release by T cells. This is in line with our recently published results on the analogous fusion protein EpCAM-ReTARG^TPR^, which redirected anti-CMV_pp65_ CTLs to selectively eliminate EpCAM-expressing solid cancer cells with comparable potency to the EpCAM-directed BiTE solitomab, though at markedly reduced T cell-secreted IFNγ levels [11].

The decoration of CD19^pos^ B cells with CD19-ReTARG^TPR^ closely mimics a CMV infection and induces the formation of a pHLA-I/TCR complex with anti-CMV CD8^pos^ T cells, which subsequently eliminate target cells. This pHLA-I/TCR interaction leads to the secretion of physiological levels of secreted cytokines, which is in contrast to cytokine levels secreted upon BiTE or CAR T cell treatment. The exact reason for such big differences in cytokine levels is yet to be elucidated but is probably caused by a combination of factors including the negative regulation of T cell activation and chronic activation. Physiological T cell activation, including that induced by CD19-ReTARG^TPR^, is tightly regulated, and the highly layered architecture of negative T cell regulation, including transmembrane adaptors (PAG and SIT), phosphatases (SHP-1 and PTEN), kinases (DGKs), and E3 ligases, prevents the overstimulation of T cells, which would result in autoimmunity or early T cell exhaustion (reviewed in [20]). CAR T cells incorporate synthetic signaling domains and are engineered to signal independently of endogenous pHLA-I/TCR interaction, which limits the effectiveness of regulatory mechanisms. In addition, these stimulatory domains are always “on”, leading to enhanced cytokine secretion. Similarly, BiTEs link T cells to cancer cells via CD3 engagement, which also causes the loss or dysregulation of the normal architecture of inhibitory regulation.

We provide compelling in vitro evidence that redirecting anti-CMV T immunity by CD19-ReTARG^TPR^ potently eliminates CD19^pos^ hematological cancer cells, without inducing supraphysiological cytokine responses, as seen during the CAR T cell and BiTE treatments used clinically. In particular, the secretion of IL6 and TNFα induced by CD19-ReTARG^TPR^ is markedly reduced compared to that in the BiTE and CAR T cell treatments. Interestingly, both IL6 and TNFα are among the core cytokines involved in cytokine release syndrome (CRS), and IL6 blockade by Tocilizumab is often employed in an attempt to relieve life-threatening aspects of CRS [21,22]. We therefore speculate that CD19-ReTARG^TPR^ would translate to improved clinical safety.

This study is an early proof-of-concept investigation focused on demonstrating the fundamental feasibility of the approach. A limitation of the current study is the lack of in vivo validation of these findings, but these experiments are in preparation and will comprise of xenografted (CD19^pos^/luciferase-expressing malignant B cell line) immunocompromised mice treated with CD19-ReTARG^TPR^, CD19-BiTE, or CD19-CAR T cells. We will compare their anticancer efficacy and determine the levels of secreted pro-inflammatory cytokines. Although mouse studies cannot fully predict whether irAEs may arise in clinical trials in humans, they should give a good indication of the functionality and safety of the ReTARG approach.

The CD19-ReTARG^TPR^ approach appears to be particularly promising for CD19-expressing leukemia, given that approximately 72% of CLL patients are CMV-seropositive and maintain functional anti-CMV T cell responses, while generalized anergy is often observed in their CD8^pos^ T cell compartments [8,23].

Despite encouraging clinical outcomes, therapeutic success with CD19-directed CAR T cell therapy is often limited due to poor engraftment, insufficient persistence, and eventual disease recurrence [12,24]. The favorable characteristics of anti-CMV T cells, including their robust cytotoxic activity, persistence, and capacity to home and remain functional in an immunosuppressive tumor microenvironment, have prompted their use in CD19-CAR T cell approaches [25]. Such strategies involve grafting the CD19-CAR construct onto expanded CMV-specific T cells (CMV-CD19-CAR T cells), thereby leveraging the long-lived nature of virus-specific memory T cells to sustain antitumor responses [26]. Moreover, this opens up possibilities for the post-infusion boosting of CMV-CD19 CAR T cells through CMV vaccination protocols [26,27]. In light of this, we speculate that the ReTARG approach could be of significant added value when applied with CMV-CD19 CAR T cells in a combinatorial strategy.

CD19 is not only expressed on B cell malignancies but plays a key role in B cell biology and is expressed during all stages of B cell development. Therefore, CD19-ReTARG^TPR^ may target healthy B cells as well. From studies in CD19 CAR T cells and BiTEs, we know that this can cause long-lasting B cell depletion and hypogammaglobulinemia [28,29]. Despite these widespread effects on the immune system, the incidence of severe infections after CAR T cell therapy is relatively low, and the prevention of serious infections can be achieved through immunoglobulin replacement [30,31,32,33].

Target antigen downmodulation is a well-documented resistance mechanism observed in both CAR T cell and BiTE therapies. Notably, CAR T cells have been shown to require a target antigen presence that is 100- to 1000-fold greater compared to T cells activated through physiological TCR engagement [34,35]. Indeed, we observed that CD19-ReTARG^TPR^ efficiently redirected anti-CMVpp65 CTLs to eliminate cancer cells with low levels of CD19, a context in which CD19 CAR T cells and blinatumomab exhibited markedly diminished activity. While the complete loss of CD19 expression remains a challenge for any CD19-directed approach, the relatively simple, modular nature of the ReTARG fusion protein design allows for rapid adaptation to alternative or additional tumor antigens. Currently, we are constructing a novel series of RETARG^TPR^ variants incorporating dual-targeting moieties, including combinations against CD20 [36], CD22 [37,38], CD38 [39], CD123 [40], and BCMA [41,42].

AICD is an essential homeostatic mechanism that ensures timely termination of immune responses following antigen clearance. CAR T cells, due to their uncontrolled tendency for non-physiological hyperactivation, are especially susceptible to AICD [17]. Indeed, co-culture of cancer cells that highly express CD19 resulted in marked AICD in CD19 CAR T cells, whereas CD19-ReTARG^TPR^-redirected anti-CMV_pp65_ CTLS achieved comparable target cell elimination with no or minimal AICD observed. Consistent with previous reports, under similar conditions, we also did not observe increased AICD in T cells redirected by blinatumomab, reinforcing that AICD is predominantly a concern for CAR T cell approaches [17,43].

Unlike CAR T cells or BiTEs, which broadly activate all CD3^pos^ T cells in an HLA-unrestricted manner, ReTARG^TPR^-mediated T cell redirection is limited to CMV-seropositive individuals expressing the common HLA-B*07:02 allele. However, HLA typing and CMV serostatus assessment are clinically routine and do not represent significant barriers to implementation. The global frequency of the HLA-B*07:02 is >5%, and CMV seroprevalence ranges from ~80% in Europe in North America to 100% in Africa and Asia [44,45]. Although CD19-ReTARG^TPR^ treatment can be applied to a large fraction of the population, patients might not have autologous T cells suitable for redirection by this particular ReTARG fusion protein (e.g., CMV seronegative and/or HLA-B*07:02 negative). As previously noted, the modular nature of the ReTARG fusion protein design allows for its straightforward adaptation to any other (common or rare) HLA-I allele by incorporating the corresponding CMV-derived peptide. Alternatively, the ReTARG approach can be adapted to exploit antiviral T cell immunity induced by natural infection and/or vaccination directed at other common viruses, including SARS-CoV2 [46], HPV [47], and EBV [48]. In addition, we recently demonstrated that ReTARG fusion proteins can be “armed” with immunostimulatory cytokines to further enhance their efficacy and/or overcome TME-induced immunosuppression [10]

To our knowledge, this research is the first to demonstrate the successful redirection of anti-CMV T cells to selectively eliminate CD19^pos^ cancer cells using a single, self-contained fusion protein. Previous approaches, such as those by Mous et al. [49] and Schütz et al. [50], relied on multi-component systems that require assembly (e.g., streptavidin-biotin coupling or nanoparticle conjugation). Such additional steps may introduce complexity and the risk of improper assembly, potentially hampering functionality. In contrast, CD19-ReTARG^TPR^ is designed as a monomeric fusion protein, simplifying manufacturing and reducing the likelihood of producing defective molecules.

## 5. Conclusions

In conclusion, CD19-ReTARG^TPR^ effectively eliminates CD19-expressing cancer cells while avoiding several major limitations of CAR T cell and BiTE therapies, including excessive cytokine release, AICD, and escape due to diminished target antigen expression. Its physiological mechanism of action, modularity, and compatibility with memory virus-specific T cells represent advances in the development of targeted, safer, and more adaptable immunotherapies for hematological malignancies.

## Data Availability

All data relevant to this study are included in the article.

## Supplementary Materials

The following supporting information can be downloaded at: https://www.mdpi.com/article/10.3390/cancers17142300/s1, Full pictures of the Western blots in Figure 1C.

## Abbreviations

The following abbreviations are used in this manuscript:

AICD	activation-induced cell death
BiTE	bispecific T cell engager
CAR	chimeric antigen receptor
CLL	chronic lymphocytic leukemia
CMV	cytomegalovirus
CRS	cytokine release syndrome
CTL	cytotoxic CD8^pos^ T cell
E:T	effector to target
ICANS	immune effector cell-associated neurotoxicity syndrome
IL2	interleukin 2
irAEs	immune-related adverse events
NR	non-reducing
PBMCs	peripheral blood mononuclear cells
pHLA-I	peptide-human leukocyte antigen class I
R	reducing
TCR	T cell receptor
TME	tumor microenvironment
TPR	TPRVTGGAM
