# Supplementary figures and images for: CD19-ReTARGTPR: A Novel Fusion Protein for Physiological Engagement of Anti-CMV Cytotoxic T Cells Against CD19-Expressing Malignancies
Source: Cancers (Basel). 2025 Jul 10;17(14):2300. doi: 10.3390/cancers17142300 (PMC12293208; doi:10.3390/cancers17142300)

CD19-ReTARG<sup>TPR</sup>  
Not reduced

CD19-ReTARG<sup>TPR</sup>  
Reduced

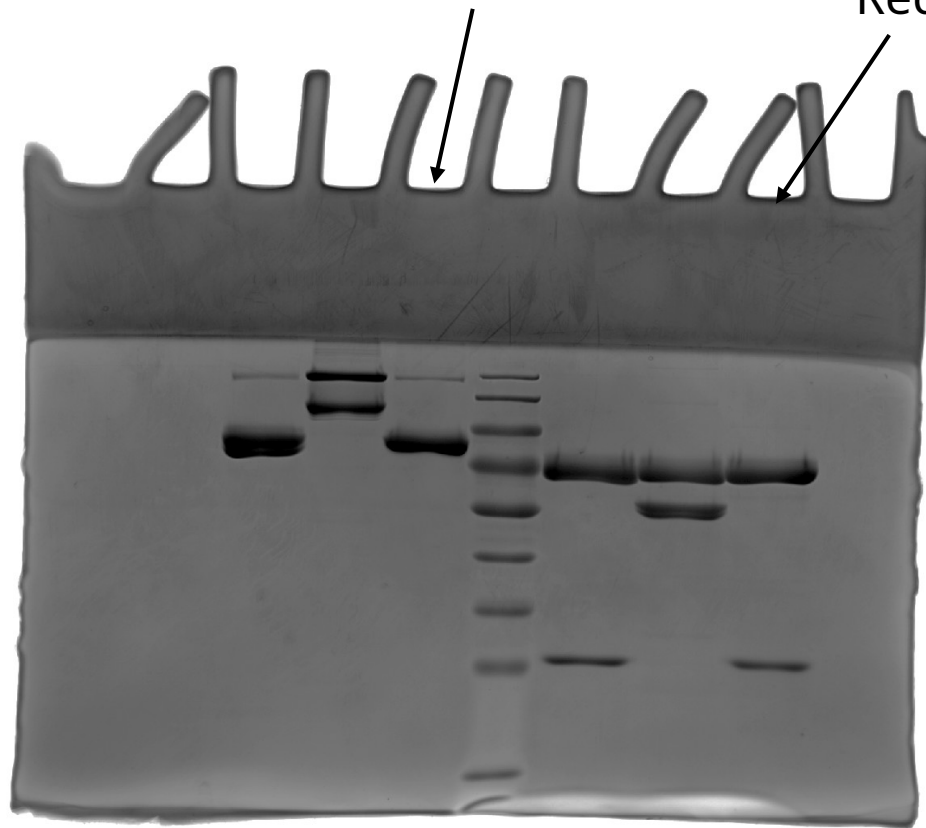

Supplement: Supplementary file 1 [file cancers-17-02300-s001.zip › cancers-3697970-supplementary.pdf]
